# Exploring Adolescents’ Perceptions of Hearing Loss and Hearing Aids in Greece: A Survey Study

**DOI:** 10.3390/audiolres15030058

**Published:** 2025-05-12

**Authors:** Ioanna Fragoulia, Nikolaos Trimmis, Voula Chris Georgopoulos

**Affiliations:** 1 Department of Speech & Language Therapy, University of Patras, 26504 Rion, Greecenicktrimmis@upatras.gr (N.T.); 2 Primary Healthcare Laboratory, School of Health Rehabilitation Sciences, University of Patras, 26504 Rion, Greece

**Keywords:** hearing loss awareness, hearing aids, adolescents’ perceptions

## Abstract

**Objectives:** This study investigates Greek teenagers’ general knowledge and perception of hearing loss, hearing aid accessibility, and the challenges associated with hearing impairment. **Methods**: A 27-item self-reported questionnaire was developed and distributed to 152 participants (aged 12–18) to assess their familiarity with hearing loss, amplification availability, communication strategies, and perceptions of hearing loss. **Results**: While 94.7% of participants recognized that hearing loss affects communication, only 10.5% correctly identified how hearing aids are accessed. Additionally, 42.1% reported having temporarily experienced some form of hearing loss. Most participants (94.7%) acknowledged communication difficulties faced by individuals with hearing loss, and only 3.4% reported being unable to communicate effectively with someone affected. Among those aware of bullying incidents involving individuals with hearing loss, 78.1% identified classmates as the primary source. **Conclusions**: These findings are consistent with international research and highlight the need for targeted education, awareness initiatives, and clearer access pathways to hearing technology. Improving adolescent hearing health literacy may facilitate early intervention, reduce stigma, and promote inclusion for peers affected by hearing impairment.

## 1. Introduction

According to data from the World Health Organization (WHO), approximately 466 million people worldwide experience hearing loss, including 34 million children. This number is projected to rise, with estimates suggesting that by 2050, over 900 million people—one in ten individuals globally—will be affected [1]. Despite advancements in medical technology, only 20% of individuals with hearing loss seek assistance, and merely 11% use hearing aids, with the average user age being 75 years [2]. Even in developed countries, hearing loss remains an underestimated health condition, primarily associated with older adults. However, hearing loss among adolescents is an increasing public health concern.

Hearing loss is recognized as the third most prevalent chronic health condition among older adults [3]. It is estimated that one in three adults aged 65–74 has significant hearing impairment that interferes with communication. Audiologists are the primary healthcare professionals responsible for assessing, diagnosing, treating, and managing hearing loss and balance disorders in both children and adults [4]. Additionally, speech–language pathologists play a crucial role in evaluating and rehabilitating individuals with hearing loss, particularly in addressing communication disorders, speech difficulties, and language processing challenges.

Effectively addressing hearing loss often requires a multidisciplinary healthcare team, which typically includes an otolaryngologist (ENT specialist), audiologist, speech-language pathologist, psychologist, social worker, and hearing aid specialist. These professionals work together to provide comprehensive care and improve the quality of life for individuals with hearing impairment [5].

While hearing loss is often associated with older adults, adolescent hearing impairment is an increasing public health concern Shargorodsky et al. [6] reported a significant rise in hearing loss prevalence among U.S. adolescents, increasing from 14.9% in 1988–1994 to 19.5% in 2005–2006. However, more recent data from Wu et al. [7] show that prevalence declined to 10.9% by 2017–2020, though the causes of this decline remain unclear. Despite this decrease, adolescent hearing loss continues to pose a public health concern due to its impact on communication, learning, and social development. This ongoing risk highlights the importance of early detection and systematic access to hearing care, in line with the WHO recommendations emphasizing early identification as key to the effective management of hearing loss in children and adolescents [1].

A leading cause of hearing loss among adolescents is noise-induced hearing loss (NIHL), often resulting from unsafe listening habits such as prolonged exposure to loud sounds. Degeest et al. [8] found that adolescents frequently engage in high-risk listening behaviors, such as prolonged exposure to loud music through personal music players, yet rarely use hearing protection. Similarly, Alnuman and Ghnimat [9] found that while 64.1% of young adults in Jordan recognized hearing loss as a significant issue, only 9.8% used ear protection, though educational interventions significantly improved their willingness to adopt preventive measures. In a study in Saudi Arabia, it was reported that adolescents frequently listen to music at dangerously high volumes, increasing their risk of NIHL [10]. Likewise, Lee and Jeong [11] found that 32.2% of high school students in South Korea reported a perceived decline in hearing due to personal listening device (PLD) use, with many exceeding daily safe noise limits. These findings highlight the urgent need for awareness campaigns and education programs to reduce the risk of NIHL among adolescents, such as the NIDCD’s ‘It’s a Noisy Planet. Protect Their Hearing’ campaign [12] and initiatives by the European Environment Agency addressing the effects of environmental noise on children [13].

In addition to hearing loss risks, adolescents often hold misconceptions about hearing aids, which contribute to low adoption rates. Many believe that hearing aids are only for the elderly, are bulky and noticeable, or that they fully restore hearing. Such misunderstandings can discourage young individuals from seeking the assistance they need [14]. Recent research suggests that adolescents may also experience inconsistent use due to social stigma, peer reactions, and practical barriers such as device management and discretion [15]. Unrecognized hearing loss in adolescents can negatively impact academic performance, peer relationships, and emotional well-being, reinforcing the need for early detection and awareness [16]. Many adolescents are exposed to harmful noise levels at school, yet hearing protection and education programs remain limited, reinforcing the need for early detection and awareness initiatives to prevent long-term hearing problems [17]

Hearing loss can also have serious social and emotional implications for adolescents. Research has shown that teenagers with hearing impairments frequently experience bullying from their peers and often struggle with anxiety and sleep disturbances [18]. Archbold et al. [19] found that children and young people with mild or moderate hearing loss in the United Kingdom often face social and emotional challenges, particularly in mainstream schools that lack sufficient deaf awareness. Similarly, a U.S. study [20] found that 34.4% of parents reported that their child had experienced bullying due to hearing loss, and 87% of adolescents with hearing impairments avoided using hearing aids out of fear of being bullied.

Further evidence of the social challenges faced by adolescents with hearing loss comes from a qualitative cohort study in the UK [21]. The study found that while most adolescents with hearing impairments had positive peer relationships, those with moderate hearing loss struggled to form friendships and often felt different from their typically hearing peers in mainstream schools. The visibility of hearing aids was a contributing factor to social integration difficulties, with girls experiencing more friendship conflicts than boys. Additionally, Bouldin et al. [18] reviewed 17 studies comparing bullying experiences among children with and without hearing loss, confirming that children with hearing impairments are at a higher risk of being bullied but are less likely to engage in bullying behavior themselves. Interestingly, bullying was not necessarily triggered by the visibility of hearing aids but was influenced by environmental and social factors, such as peer interactions, school settings, and parental support.

Access to hearing healthcare and awareness of hearing loss can vary significantly between urban and rural populations. Research indicates that hearing loss prevalence is generally higher in rural areas, often due to increased occupational and environmental noise exposure, as well as limited healthcare access [22]. This has also been shown for adolescents living in rural areas in the US [23]. Given these disparities, it is important to explore whether teenagers’ knowledge and perceptions of hearing loss and hearing aids differ based on their place of residence. The place of residence (urban vs. rural) can influence exposure to environmental noise, accessibility to audiological services, and community attitudes toward disability. Understanding how location shapes knowledge may help address inequalities in hearing healthcare access and awareness. Recent findings further underscore this issue, with studies reporting persistent rural disparities in adolescent hearing health [24].

The present study aims to investigate Greek teenagers’ knowledge and perceptions regarding hearing loss and hearing aids. Specifically, it examines whether knowledge levels differ based on sex, personal acquaintance with someone who has hearing loss, and place of residence (urban vs. rural areas). It also explores whether the educational level (Gymnasium [Grades 7–9] vs. Lyceum [Grades 10–12]) influences teenagers’ awareness and understanding. Additionally, this study intends to provide data that can inform school-based awareness initiatives and public health strategies targeted at adolescents. Moreover, it highlights the importance of addressing bullying, early detection, and the social stigma associated with hearing loss—factors which may support a better uptake of assistive technology and promote peer inclusion.

This study does not aim to evaluate hearing aid usage among adolescents with hearing impairment, but rather to investigate general adolescent awareness, attitudes, and experiences regarding hearing loss and assistive technology.

To meet its objectives, this study explored the following research questions: (1) What is the level of knowledge among adolescents about hearing loss and hearing aids? (2) How do demographic factors (e.g., sex, place of residence, education level) influence adolescents’ perceptions of hearing loss and assistive technology? (3) How do adolescents communicate with peers who have hearing loss? (4) Is there a perceived link between hearing loss and bullying among adolescents?

The following sections outline the research methods, including participant selection, data collection, and analysis procedures.

## 2. Materials and Methods

This study utilized a 27-item questionnaire, developed using Google Forms, to explore teenagers’ knowledge and perceptions regarding hearing loss and hearing aids. The questionnaire aimed to assess general knowledge, awareness of hearing aid accessibility, and personal experiences related to hearing loss. The full English version of the questionnaire is provided in Appendix A.

This study targeted adolescents, following the World Health Organization’s (WHO) definition of adolescence as individuals aged 10 to 19 years. However, for this study, participants were limited to teenagers aged 12 to 18 years, corresponding to students from Lower Secondary School (Gymnasium; Grades 7–9) to Upper Secondary School (Lyceum; Grades 10–12) in Greece. Apart from the age requirement, no additional inclusion or exclusion criteria were applied.

The 27-item questionnaire was developed specifically for this study, drawing on themes identified in the prior literature and public awareness campaigns concerning adolescent hearing health. It was reviewed for content relevance and age appropriateness by the second author, who is a dually certified audiologist and speech–language pathologist. Item wording was chosen to ensure accessibility and comprehension for the adolescent population. Questions were phrased using non-technical, everyday language and were reviewed for clarity by both a clinical audiologist and a speech–language pathologist. Correct answers for knowledge-based items were defined during questionnaire development based on a consensus between a licensed audiologist and a speech–language pathologist, ensuring alignment with current hearing health guidelines. The questionnaire was then pilot tested with six adolescents (three from Gymnasium and three from Lyceum). During pilot testing, participants were encouraged to provide feedback on terminology and comprehension, which led to minor changes in wording (e.g., replacing ‘hearing impairment’ with ‘hearing difficulty’).

Questions regarding hearing aid access and provider qualifications were included to assess adolescents’ understanding of how hearing care is regulated. In Greece, as in many countries, hearing aids are classified as medical devices that must be dispensed by trained professionals. However, the increasing availability of unregulated amplifiers through retail and online platforms can create confusion. Given that adolescents often rely on social media for health-related information, their ability to distinguish between credible and informal sources is especially important [25].

Participants were recruited using snowball sampling, primarily through school and parent networks. Parents were first contacted directly by the researchers and were provided with study information and consent forms. Only after obtaining written parental consent were adolescents invited to participate. Adolescents then provided their own informed assent before completing the questionnaire. To ensure participant privacy, no identifying personal data (such as names or contact information) were collected, and responses were fully anonymous. Sex, age, and place of residence were treated as non-sensitive demographic variables within the scope of anonymized survey research. The questionnaire was administered electronically between May and June 2023.

Basic demographic information was collected, including sex, age, place of residence (urban or rural), and type of school attended (middle school or high school). These demographic data were used to categorize responses and analyze potential differences in knowledge and attitudes toward hearing loss based on sex, place of residence, and school level. Hearing loss status was self-reported by participants and was not clinically verified; responses reflect the adolescents’ personal understanding of their hearing experience, whether temporary or perceived.

Descriptive statistics were used to summarize participant demographics and item response frequencies. Chi-square (χ^2^) tests were used to explore associations between demographic variables and questionnaire responses. All statistical analyses were conducted using IBM SPSS Statistics version 27 (IBM Corp., Armonk, NY, USA) [26], with significance set at *p* < 0.05.

## 3. Results

A total of 162 participants took part in this study, but 10 were excluded for not meeting the age criteria, resulting in a final sample of 152. Participants had a mean age of 16.26 ± 1.5 years (range 12–18 years). Table 1 summarizes the demographic characteristics of the participants. Overall, there were more female participants than male, most participants attended Lyceum rather than Gymnasium, and slightly more participants lived in rural areas than in urban areas.

A total of 108 out of 152 participants reported knowing someone with hearing loss, while 2 participants were hearing impaired. Among those who knew someone with hearing loss, 63 participants reported encountering them most frequently in their family environment, 53 in their social environment, and 39 in their school environment, with the latter being the least common setting.

A total of 32 participants reported that the individuals they knew with hearing loss had experienced bullying. Only participants who reported knowing someone with hearing loss were asked whether that individual had experienced bullying. Therefore, the responses regarding bullying awareness reflect only those with direct exposure to individuals with hearing impairment. The majority (78.1%) identified classmates as the primary source of bullying, followed by friends (25%). Additionally, two participants (6.3%) stated that bullying came from family members, while four participants (12.5%) selected “other”, describing bullying as coming from society or strangers on the street. Notably, no participants reported bullying by teachers.

The majority of participants (125 out of 152) correctly identified that hearing loss is more prevalent among older adults. Regarding personal experience, 113 participants (74.3%) had interacted with someone with hearing loss at least once in their lives, while 39 (25.7%) had not.

Regarding communication strategies, 94 participants (83.2%) reported raising their voice to improve understanding. Additionally, 46 participants (40.7%) deliberately faced the person to facilitate lip reading, while 36 (31.9%) used gestures and facial expressions. Fewer participants relied on written communication (6.2%), and only four (3.4%) reported being unable to establish a dialogue with a person with hearing loss.

When asked about personal hearing loss, 64 (42.1%) reported having experienced it at some point in their lives. Since multiple responses were allowed, otitis was the most frequently selected cause (60.9%), followed by loud music exposure (43.8%) and environmental noise (34.4%). Other selected causes included surgical procedures (6.3%) and miscellaneous factors, such as exposure to jackhammers or seawater (9.4%).

Only 36 participants (23.7%) correctly stated that two hearing aids are required for individuals with hearing impairment. Notably, just one participant selected the response that hearing aids are unnecessary since sign language can be learned. This reinforces the widespread understanding that hearing aids are the primary treatment for individuals with hearing loss. Interestingly, the participant who selected this response also reported knowing people with hearing loss in multiple environments, including school, family, and social settings. This suggests that their perspective may have been shaped by firsthand experiences with individuals who primarily communicate using sign language.

This question was presented in the form of a multiple-choice item (Question 16: ‘Select the correct statement from the options below’). One of the response options stated that ‘two hearing aids are required for a person with hearing impairment’. While the correct answer assumed bilateral hearing loss, this was not explicitly stated in the question. We acknowledge that this may have influenced participant responses, and we plan to revise the question wording in future versions of the survey to ensure greater clarity.

Only 15 participants (10.5%) correctly answered that hearing aids can be purchased from a specialized hearing aid store. Sixty-one participants (40.1%) incorrectly responded that hearing aids can be obtained from a medical office, while 52 participants (34.2%) believed they could be purchased at a pharmacy. Additionally, 13.8% stated that hearing aids could be found at an optical store. The responses deemed correct in this study were evaluated based on the policies, practices, and healthcare system currently in place in Greece. It is recognized that certain answers might be considered correct in other countries due to differences in legislation, hearing aid distribution systems, or clinical practices. These contextual factors were not included in the questionnaire but should be considered when interpreting cross-cultural comparisons.

Participants identified various factors as potential causes of hearing loss. The most frequently selected cause was heredity, chosen by 99 participants (65.1%), followed closely by exposure to noisy environments, reported by 83 participants (54.6%). Additionally, infections (such as otitis) and medication use were acknowledged by 77 participants (50.7%) as contributing factors. In contrast, fewer participants associated hearing loss with complications during pregnancy or birth (36 participants, 23.7%), poor dietary habits (11 participants, 7.2%), or were unsure of the causes (29 participants, 19.1%). These responses highlight a general awareness of environmental and genetic influences on hearing loss, while less emphasis was placed on nutritional and prenatal factors.

The vast majority of participants (144 out of 152, or 94.7%) correctly acknowledged that individuals with hearing loss face communication challenges. When asked about the impact of hearing aids on quality of life, the most common response was that they improve it “considerably”, with 66 participants (37.5%) supporting this view. This suggests a general awareness of the benefits of hearing aids, though the perceptions of their effectiveness varied among respondents.

The majority of participants (63.2%) correctly stated that a child with hearing loss can attend a mainstream school. However, 25% were unsure, while 11.8% believed that a child with hearing loss could not enroll in a typical school.

Regarding an awareness of support groups for hearing loss, opinions were almost evenly split: 52% (79 participants) reported not knowing whether such groups exist, whereas 48% (73 participants) were aware of their existence. This indicates a relatively balanced level of awareness, suggesting the need for greater public information on available support resources.

A Chi-square test of independence was performed to examine the relationship between various factors and the knowledge of hearing loss and hearing aids. The results were as follows: No statistically significant difference was found for sex, χ^2^(5, N = 150) = 8.56, *p* = 0.128, or for place of residence (urban vs. rural), χ^2^(5, N = 152) = 7.20, *p* = 0.206. Additionally, no statistical difference was found for educational level (Gymnasium vs. Lyceum), χ^2^(5, N = 152) = 2.57, *p* = 0.766.

**Figure 1:**
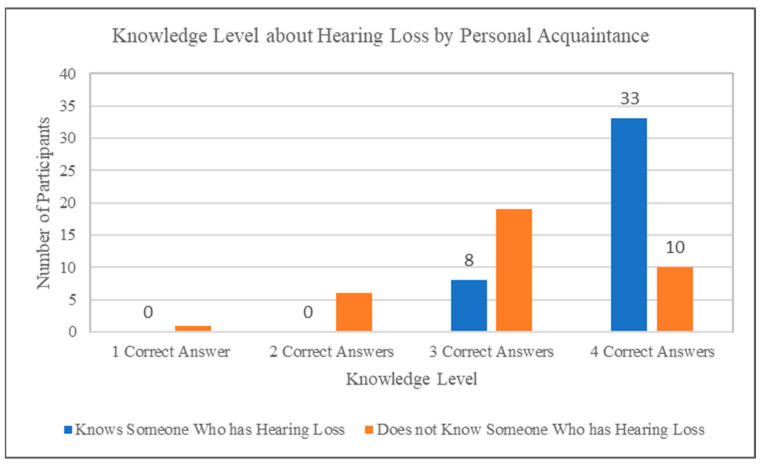
Relationship between participants’ personal acquaintance with someone who has hearing loss and their performance on knowledge-based survey items. Knowledge levels are represented by the number of correct answers across items designed to assess the understanding of hearing loss and hearing aids, based on expert-defined responses.

A significant relationship was observed between adolescents’ personal acquaintance with someone experiencing hearing loss and their knowledge about hearing loss and hearing aids (χ^2^(4, N = 152) = 58.74, *p* < 0.001). As illustrated in Figure 1, participants who personally knew someone with hearing loss generally demonstrated higher knowledge levels, achieving more correct answers compared to participants without personal acquaintance.

A Chi-Square Goodness of Fit test was conducted to determine whether the distribution of responses (Yes/No) to the question of whether the individual had been bullied differed significantly from a 50–50 distribution. The results indicated a significant difference, χ^2^(1, N = 110) = 19.236, *p* < 0.001. Specifically, there were more “No” responses (78) than expected (55) and fewer “Yes” responses (32) than expected (55), suggesting that knowledge of bullying was not equally distributed across the two categories.

As multiple Chi-square tests were conducted without a statistical correction for multiple comparisons, these analyses should be considered exploratory and interpreted with caution due to the increased risk of a Type I error.

## 4. Discussion

This study explored Greek adolescents’ knowledge and perceptions of hearing loss, hearing aids, and bullying, providing key insights into their awareness of hearing impairment, interactions with individuals with hearing loss, and experiences with bullying among those affected.

Hearing loss was a familiar condition for most respondents, with 71.1% reporting that they knew someone with hearing loss. Encouragingly, a high percentage of participants demonstrated accurate knowledge in key areas: 82.2% recognized that hearing loss is more common among the elderly, 94.7% acknowledged that individuals with hearing loss face communication difficulties, 86.2% were aware that selling hearing aids in Greece requires specialized knowledge, and 63.2% correctly stated that a child with hearing loss can attend a mainstream school. However, gaps in knowledge were also evident. When asked where hearing aids can be obtained, only 10.5% correctly identified specialized hearing aid stores, highlighting a limited awareness of hearing aid accessibility. While such information may be more directly relevant to parents, educators, or healthcare professionals, adolescents’ awareness of how hearing aids are obtained and regulated can shape their perceptions of accessibility and normalization of assistive technology. These insights are important for understanding broader patterns of hearing health literacy and the social context in which adolescents support peers or seek help themselves.

When comparing responses between Gymnasium and Lyceum students, minor but notable differences were observed. For instance, only 6.5% of Gymnasium students correctly identified how hearing aids are accessed, compared to 10.7% of Lyceum students. An awareness of hearing loss was also slightly higher among Lyceum students (72.7%) than Gymnasium students (64.5%). These patterns suggest that older adolescents may have slightly more exposure to or understanding of hearing health issues, possibly due to cognitive maturity, curriculum exposure, or life experience. Such age-related differences may warrant further exploration in future research and should be considered when designing age-appropriate awareness programs.

### 4.1. Bullying and Social Challenges

Bullying was reported by 29% of participants who knew someone with hearing loss. Among these, 78.1% identified classmates as the primary source, followed by friends (25%). These findings are broadly consistent with [18], who reported a direct link between hearing loss and bullying in seven out of seventeen studies. Both sources emphasize that classmates are the primary perpetrators of bullying among adolescents with hearing loss.

Although not statistically significant, slightly more Gymnasium students (37.5%) than Lyceum students (26.7%) reported an awareness of the bullying experienced by individuals with hearing loss. This trend may reflect differences in peer group dynamics or the varying exposure to anti-bullying initiatives across educational levels. While our dataset does not show a conclusive pattern, prior research suggests that peer bullying is more common among younger age groups, particularly in primary and early secondary school. As students mature, the nature of bullying tends to shift from physical aggression to more verbal and psychological forms, which may have more significant impacts on mental health and self-esteem [27]. These insights underscore the need for age-specific interventions: younger students may benefit from early screening and subtle sign awareness, while older students may require support structures addressing stigma, emotional resilience, and social integration.

Warner-Czyz et al. [28] found that children with hearing loss are at an increased risk of peer victimization, often experiencing social exclusion and teasing. Our study did not assess bullying risk directly; however, participants who knew individuals with hearing loss frequently identified classmates as the primary source of bullying. This aligns with previous findings on peer-related social challenges. These results highlight the need for comprehensive support systems and anti-bullying programs within educational settings to foster inclusive environments for students with hearing impairments. Although the survey did not measure participants’ emotional concern regarding bullying, the data reveal that the individuals most commonly identified as bullies, classmates, and friends are typically the closest social contacts of adolescents. This finding raises concerns about the vulnerability of adolescents with hearing loss within their immediate peer environments and underscores the importance of early social awareness and school-based intervention. In addition to emotional and social consequences, bullying may also lead to practical issues, such as the accidental or intentional damage of hearing aids. These incidents can result in financial burdens or legal concerns for families and schools, further highlighting the need for protective policies and awareness among school staff.

Despite progress in inclusive education, misconceptions persist. While 63.2% of participants correctly stated that a child with hearing loss can attend a mainstream school, 25% were unsure, and 11.8% believed it was not possible. This uncertainty suggests that while integration efforts have improved, further education on accessibility, inclusion, and the academic potential of students with hearing loss is needed. However, beyond academic inclusion, social integration remains a challenge for individuals with hearing loss.

This is evident in the experiences of hearing-impaired adults in Greece, who report difficulties in forming friendships during their primary and secondary school years [29]. These challenges align with our study’s findings, particularly regarding the prevalence of bullying and social isolation among adolescents with hearing loss. A key finding from Martzos et al. [30] is that Greek deaf individuals frequently use social networking sites (SNSs) as a tool for communication and social integration, helping them connect with both deaf and hearing communities. This suggests that digital platforms might also serve as a support system for adolescents with hearing loss, particularly those facing social isolation or bullying. Given the prevalence of bullying in this study, SNSs could offer a space where teenagers with hearing loss can share experiences, access supportive communities, and engage with advocacy groups that promote hearing inclusivity.

To address these issues, schools should implement hearing health programs that integrate interactive lessons on hearing protection, hearing aid accessibility, and communication strategies. Additionally, technology-based interventions, such as smartphone applications, could help monitor safe listening habits and educate adolescents about noise-induced hearing loss (NIHL) [31]. Public awareness campaigns led by the government and health organizations should also ensure that teenagers have clear, accessible information on hearing loss prevention and available healthcare services.

Given the growing body of research suggesting a link between hearing loss and social challenges, such as peer bullying and social exclusion [18,20,27], it is important to implement school policies that support students with hearing impairments. These efforts could help create a more informed and supportive environment for adolescents at risk of or living with hearing loss.

### 4.2. Noise-Induced Hearing Loss (NIHL) and Prevention

A significant portion of participants (42.1%) reported experiencing some degree of hearing loss at some point in their lives. While otitis was the most commonly reported cause (60.9%), many participants also identified loud music exposure (43.8%) and environmental noise (34.4%) as contributing factors. Since multiple causes could be selected, these findings highlight concerns about adolescents’ exposure to unsafe noise levels and their increased risk of NIHL.

While the present study did not collect data on the duration of personal listening device use, the existing literature highlights its importance. The risk of NIHL is compounded by both high volume and extended listening duration through personal devices. Studies show that prolonged exposure—even at moderate levels—can have cumulative effects on adolescent hearing health due to daily listening time and unsafe volume practices [11]. Future research should incorporate listening duration as a variable to better assess behavioral risk factors for NIHL.

This concern is further supported in a study in South Korea that found that adolescents frequently used earphones in noisy environments, leading to an increased risk of self-reported hearing problems [32]. Similarly, Serra et al. [33] noted that adolescents often engage in high-noise activities, such as listening to personal music players at unsafe volumes, making them particularly vulnerable to NIHL.

Beyond behaviors, an awareness of NIHL risks remains low, as seen in Crandell et al. [34], who found that young adults from diverse racial and ethnic backgrounds often lacked knowledge about hearing loss prevention and had low usage rates of hearing protection devices. Their study underscores the need for targeted educational interventions to improve hearing conservation practices among youth.

Similarly, a study conducted in India found that while some adolescents demonstrated a strong knowledge about hearing impairment, a substantial percentage had limited awareness [35]. Compared to our study, where knowledge gaps were particularly evident in hearing aid accessibility and NIHL prevention, these results suggest that misconceptions about hearing health persist across different cultural and geographic settings. This highlights the universal need for school-based educational interventions aimed at improving hearing conservation awareness and access to reliable information on hearing health.

While this study focuses on Greek adolescents, Table 2 compares its findings with those of international studies to illustrate how similar challenges related to hearing loss and hearing aid perceptions are observed across different cultural and geographic contexts. Common trends are observed across key areas such as misconceptions about hearing aids, the low use of hearing protection, and the social dimensions of hearing loss.

Despite the well-documented risks of NIHL, many teenagers fail to recognize hearing loss as an immediate concern, reinforcing the need for structured hearing conservation programs in schools. In addition to prevention, awareness campaigns should also tackle the stigma associated with hearing loss, particularly as 78.1% of reported bullying cases in this study were attributed to classmates. By educating not only those at risk but also their peers and educators, schools can foster a more inclusive and supportive environment for individuals with hearing impairments.

### 4.3. Limitations

While this study provides valuable insights, certain limitations should be acknowledged.

First, although this study specifically targeted Greek students and included participants from both urban and rural areas through snowball sampling, it was not designed as a stratified or representative nationwide survey. While this approach allowed access to diverse regions, the non-randomized, self-selecting nature of snowball sampling may limit the generalizability of the results. While no significant differences were found between urban and rural participants, factors such as socioeconomic background, educational settings, and access to healthcare resources could still influence knowledge and attitudes. Socioeconomic status, in particular, was not directly measured in this study and may represent an important factor influencing adolescents’ awareness of hearing loss and hearing aid access. Future research could explore how these variables impact adolescents’ understanding of hearing loss.

Second, this study relied on self-reported data, which may be influenced by participants’ perceptions, memory recall, or social desirability bias. Their responses might not fully reflect their actual knowledge, experiences, or attitudes toward hearing loss. Additionally, while 42.1% of participants reported having experienced hearing loss at some point in their lives, this does not necessarily indicate permanent or current hearing impairment. Some participants may have recalled temporary hearing issues due to factors such as ear infections, noise exposure, or other reversible conditions. Additionally, although tinnitus is a common auditory condition among adolescents, it was not assessed in this study and may be considered in future research.

Finally, while the questionnaire covered general knowledge, attitudes, and experiences, it did not explore the psychological impact of hearing loss, stigma, or social–emotional well-being in depth. Future research should examine these aspects to develop better support strategies for adolescents with hearing impairments.

As the questionnaire was developed specifically for this study and has not undergone formal psychometric validation, the findings should be interpreted as exploratory. Further validation is needed to ensure the reliability and generalizability of the results.

## 5. Conclusions

This study highlights Greek adolescents’ knowledge and perceptions regarding hearing loss, hearing aids, and associated social challenges such as bullying. While participants demonstrated an awareness of hearing loss prevalence and communication difficulties, significant gaps emerged in understanding hearing aid accessibility and preventive strategies for noise-induced hearing loss (NIHL). These findings align with trends reported in international research, where similar knowledge gaps and stigma issues have been observed among youth populations in other countries [8,18,28,35]. This underscores the universal need for early educational interventions aimed at promoting hearing health literacy.

School-based awareness campaigns, hearing protection education, and peer sensitivity programs are vital for reducing stigma and fostering inclusive environments [31,33]. In particular, given the high rates of bullying reported by participants, policies and anti-bullying strategies must be tailored to address the specific experiences of adolescents with hearing impairments [18,28].

Additionally, raising awareness among educators and caregivers can improve early detection and referral for hearing care. Public health strategies should also consider incorporating mobile health apps and social media tools to better engage adolescents in proactive hearing conservation [30,31].

Future research should investigate the long-term psychological and social impacts of hearing loss in youth and assess the effectiveness of preventive and support programs in schools. Studies involving larger, more systematically sampled populations would also be valuable in confirming these findings and guiding data-driven policy development.

## Figures and Tables

**Table 1:** Demographic characteristics of participants (N = 152).

Characteristic	Category	N	%
Sex	Male	53	34.9%
	Female	97	63.8%
	Not Disclosed	2	1.3%
Education Level	Gymnasium (Grades 7–9)	31	20.4%
	Lyceum (Grades 10–12)	121	79.6%
Place of Residence	Urban Area	68	44.7%
	Rural Area	84	55.3%
Personal Experience with Hearing Loss (temporary)	Yes	64	42.1%
	No	88	57.9%
Acquainted with Someone with Hearing Loss	Yes	108	71.1%
	No	44	28.9%

**Table 2:** Comparison of key findings with international studies.

Theme	Present Study (Greece)	Comparable International Findings
Misconceptions about hearing aids	42% believe hearing aids fully restore hearing	Misconceptions about full restoration (UK) [19]
Use of hearing protection	10% report using ear protection	9.8% reported use of ear protection (Jordan) [9]
Awareness of bullying	30% are aware that individuals with hearing loss may be bullied	87% of adolescents avoided hearing aids due to bullying (US) [28]
Perceived link between PLD use and hearing loss	50% are aware of the risk from personal listening devices	32.2% self-reported hearing decline due to PLD use (Republic of Korea) [11]

## Data Availability

The datasets of the current study are not publicly available. The data that support the findings of this study are available on request from the corresponding author.

## Abbreviations

The following abbreviations are used in this manuscript:

ENT	Ear, nose, and throat specialist (otolaryngologist)
NIHL	Noise-induced hearing loss
PLD	Personal listening device
SNS	Social networking site
WHO	World Health Organization

## Appendix A

The 27-item questionnaire used in this study, translated into the English language, is presented below.

### What Do Teenagers Know About Hearing Loss and Hearing Aids?

The objective is to explore adolescents’ knowledge regarding hearing loss and the use of hearing aids. The questionnaire is addressed to individuals aged 12–18. All responses are anonymous, and the estimated time for completion is 5–10 min. All questions with an asterisk are required.

Note: There is a difference between the terms hearing loss and deafness.

- Hearing loss refers to partial loss of hearing, while
- Deafness refers to complete loss of hearing.

1. Sex*

☐ Male   ☐ Female   ☐ Prefer not to say

2. Age*

[Free response]

3. Level of education*

☐ Gymnasium  ☐ Lyceum

4. Place of residence*

☐ Urban area  ☐ Rural area

5. How common do you think hearing loss is?*
  (Scale: Not at all = 1–Very common = 5)

☐ 1 ☐ 2 ☐ 3 ☐ 4 ☐ 5

6. Do you know someone in your family or social circle (friends, acquaintances) who has a hearing impairment (hearing loss)?*

☐ Yes    ☐ No

7. If yes, in which context?

☐ School   ☐ Family   ☐ Social circle

8. Do you know if this person has experienced bullying because of their hearing problem?

☐ Yes    ☐ No

9. If yes, who bullied them?

☐ Family

☐ Classmates

☐ Teachers

☐ Friends

☐ Other: [Specify]

10. In which age group do you think hearing loss is more common?*

☐ Children

☐ Adults

☐ Elderly

11. Have you ever interacted with someone who had a hearing impairment?*

☐ Yes    ☐ No

12. If yes, how did you communicate with them?

☐ I spoke louder to be better understood

☐ I faced them so they could lip-read

☐ I used gestures and facial expressions

☐ I communicated in writing

☐ I could not communicate effectively

13. Have you ever temporarily lost your hearing?*

☐ Yes    ☐ No

14. If yes, what was the cause?

☐ Ear infection

☐ Surgery

☐ Loud music

☐ Environmental noise

☐ Other: [Specify]

15. Which specialist(s) do you think are responsible for treating hearing disorders?*

☐ Speech and language therapist

☐ Audiologist

☐ Occupational therapist

☐ ENT doctor

☐ Psychologist

16. Choose the correct statement from the following:*

☐ One hearing aid is enough for someone with hearing loss

☐ Two hearing aids are required for someone with hearing loss

☐ Hearing aids are not necessary if the person learns sign language

☐ All of the above

☐ I don’t know

17. Which of the following can cause hearing loss?*

☐ Noisy environments

☐ Hereditary factors

☐ Infections (e.g., otitis) and medication use

☐ Poor nutrition

☐ Complications during pregnancy or childbirth

☐ I don’t know

18. Which of the following environments do you think negatively affect hearing due to noise?*

☐ Club

☐ Stadium

☐ Schoolyard

☐ Park

☐ Concert

☐ Busy street

19. How much do you think hearing loss affects communication in everyday life?*
  (Scale: Not at all = 1–Very much = 5)

☐ 1 ☐ 2 ☐ 3 ☐ 4 ☐ 5

20. Do people with hearing loss experience communication difficulties?*

☐ Yes    ☐ No

21. In your opinion, how much can hearing aids improve the quality of life for someone with hearing loss?*

☐ Not at all

☐ Slightly

☐ Moderately

☐ Quite a bit

☐ Very much

☐ I don’t know

22. Where do you think hearing aids can be purchased?*

☐ Pharmacy

☐ Optical store

☐ Medical clinic

☐ Other: [Specify]

23. Do you think someone needs special training to sell hearing aids in Greece?*

☐ Yes    ☐ No   ☐ I don’t know

24. Do hearing aids allow people with hearing loss to hear like people without hearing loss?*

☐ Yes    ☐ No   ☐ I don’t know

25. Can a child with hearing loss attend a mainstream school?*

☐ Yes    ☐ No   ☐ I don’t know

26. Are you aware of support groups for individuals with hearing loss?*

☐ Yes    ☐ No   ☐ I don’t know

27. You may optionally use the space below to share any comments, observations, or additional information about your experiences or knowledge regarding hearing loss and hearing aids.

[Free response]
